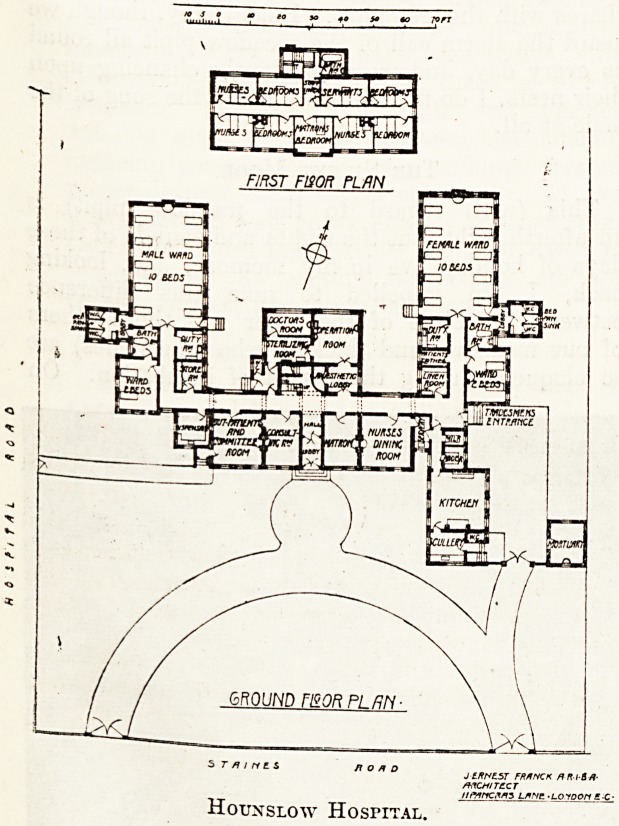# Hounslow Hospital, New Buildings

**Published:** 1913-04-19

**Authors:** 


### HOSPITAL ARCHITECTURE AND CONSTRUCTION.

### Hounslow Hospital, New Buildings.

This new building, which was opened early in January 1913, occupies a site of one acre and a third on the north side of the Staines Road. As will be seen from the plan, the main front faces south, and the axis of the large wards is north and south.

The total accommodation for patients is twenty-four beds, arranged in two wards for the male patients and two for female patients, one ward for each sex containing ten beds and the other two beds. Following the example set in the Bolingbroke Hospital, the sanitary offices are placed

Hounslow Hospital.

at the entrance end of the large wards and are thus rendered accessible to both wards. The bath rooms also are approached directly from both wards instead of being projected out with a cut-off lobby, a sensible departure from stereotyped custom.

The only means of warming the large wards apparent from the plan is the fireplace at the extreme end; it is to be presumed that hot-water radiators are installed as well, otherwise the heating power would be obviously insufficient. It rather looks as if the chief means of warming was by radiators, and the fireplace added for appearance sake. If so, it is much to be regretted; a far better plan would have been to have had two central open fireplaces of sufficient capacity to warm the whole ward, and to have provided radiators to supplement the fireplaces when required.

The operation department is arranged in a projecting wing between the wards, and comprises an anæsthetic lobby, operation room, sterilising room, and doctors' room. The precise reason for the w.c. just outside the sterilising room is not apparent, but it is in an unfortunate position and would have been better omitted.

In the main front block a small out-patient department, consisting of a waiting room and a consulting room, is provided; and a dispensary which serves both in- and out-patients.

The remainder of the block is occupied by the matron's sitting room, and nurses dining room with small servery. The kitchen offices are in a wing projecting to the south. A small mortuary is placed on the eastern boundary.

Subject to the points referred to above, the hospital is well and economically planned and shows knowledge of modern requirements.

The exterior is simple but well designed, a sound architectural effect being obtained without extravagance.

The cost amounted to £5,500, being at the rate of slightly under £230 per bed.

The architect under whom the work was carried out is Mr. J. Ernest Franck, A.R.I.B.A.

## Figures and Tables

**Figure f1:**